# Cathepsin S acts via protease-activated receptor 2 to activate sensory neurons and induce itch-like behaviour

**DOI:** 10.1016/j.ynpai.2019.100032

**Published:** 2019-05-02

**Authors:** Keshi Chung, Thomas Pitcher, Andrew D. Grant, Ellen Hewitt, Erik Lindstrom, Marzia Malcangio

**Affiliations:** a Wolfson Centre for Age-Related Diseases, King’s College London, UK; b Medivir AB, Huddinge, Sweden

**Keywords:** Itch, Proteases, Sensory neurons

## Abstract

Chronic itch is a debilitating condition characterised by excessive scratching and is a symptom frequently reported in skin diseases such as atopic dermatitis. It has been proposed that release of the cysteine protease Cathepsin S (CatS) from skin keratinocytes or immune cells resident in or infiltrating the skin could act as a pruritogen in chronic itch conditions. CatS is known to activate protease-activated receptor 2 (PAR2). We therefore hypothesised that enzymatic activation of neuronally expressed PAR2 by CatS was responsible for activation of sensory neurons and transmission of itch signals. Intradermally-injected human recombinant (hr)-CatS or the PAR2 agonist, SLIGRL-NH_2_ behaved as pruritogens by causing scratching behaviour in mice. Hr-CatS-induced scratching behaviour was prevented by CatS inhibitors and PAR2 antagonists and reduced by 50% in TRPV1^−/−^ mice compared with wild-type mice, whilst no significant reduction in scratching behaviour was observed in TRPA1^−/−^ mice. Cultured dorsal root ganglion (DRG) cells showed an increase in [Ca^2+^]_i_ following incubation with hr-CatS, and the percentage of neurons that responded to hr-CatS decreased in the presence of a PAR2 antagonist or in cultures of neurons from TRPV1^−/−^ mice. Taken together, our results indicate CatS acts as a pruritogen via PAR2 activation in TRPV1-expressing sensory neurons.

## Highlights

• Intradermal injection of cathepsin S induces scratching behaviour in mice.
• Cathepsin S inhibitors prevent cathepsin S-induced itch.
• Cathepsin S acts as a pruritogen via protease-activated receptor 2(PAR2) in TRPV1-expressing neurons.
• Cathepsin S-induced itch can be used as translational model and for testing new indications for cathepsin S inhibitors.

## 1 Introduction

Itch is defined as an unpleasant sensation in the skin associated with the urge to scratch (Steinhoff et al., 2006). Acute itch is a beneficial sensation that encourages scratching to remove irritants and potentially harmful agents from the skin (Green and Dong, 2016, Hoon, 2015, Ross, 2011). However, in chronic itch conditions such as atopic dermatitis (AD), excessive scratching of the skin results in inflammation and exacerbates the itch, culminating in an “itch-scratch cycle” (Steinhoff et al., 2006). Anti-histamines have been used to treat itch, although they are reported to have limited efficacy in AD (Andersen et al., 2017, Klein and Clark, 1999, Rukwied et al., 2000, Wahlgren et al., 1990, Yarbrough et al., 2013). It has thus been suggested that itch in AD is due to pruritogens other than histamine acting on sensory neurons. The sensory neurons that convey itch signals, or pruriceptors, mostly belong to a subset of nociceptors expressing the TRPV1 receptor (Liu and Ji, 2013). Neurons that respond to pruritogens other than histamine are proposed to additionally express the TRPA1 receptor, and it is likely that the neurons involved in AD and other chronic itch conditions belong to this population of pruriceptors (Bautista et al., 2013, Wilson et al., 2011).

Following activation by pruritogens, neurotransmitters such as glutamate and neuropeptides such as natriuretic polypeptide B are released from the central terminals of pruriceptors in the outer laminae of the spinal cord dorsal horn (Green and Dong, 2016, Hoon, 2015). The signal is then transmitted through a series of interneurons where the signal may become modified, for instance by incoming signals from nociceptive neurons that can inhibit transmission of pruriceptive information by interacting with the itch circuitry. From the projection neurons in lamina I of the dorsal horn, the neurons conveying pruriceptive signals decussate and ascend along the spinothalamic tract to the thalamus and higher brains centres, where the itch sensation is perceived (Hoon, 2015).

Besides histamine-sensitive fibres, itch is generated by recruitment of cutaneous nerve fibres that respond to mast cell derived-tryptase and activation of protease-activated receptor 2 (PAR2). Recent observations in healthy human subjects indicate that PAR2 receptor-mediated itch can also be elicited by the cysteine protease cathepsin S (CatS): human recombinant CatS (hr-CatS) applied to human skin can induce itch within minutes (Reddy et al., 2010). In addition, hr-CatS activates PAR2 receptors heterologously expressed in HeLa cells (Elmariah et al., 2014). CatS is not normally expressed in keratinocytes, but it is up-regulated in inflammatory conditions such as AD (Schonefuss et al., 2010). Consistently, CatS-overexpressing transgenic mice develop a skin disorder similar to chronic AD (Kim et al., 2012). Cleavage of PAR2 by proteases results in the sensitisation of TRP channels, including TRPV1 and TRPA1 (Amadesi, 2004, Amadesi et al., 2006, Chen et al., 2011, Dai, 2004, Dai et al., 2007, Mrozkova et al., 2016), which may be responsible for the transmission of itch signals in pruriceptors. CatS causes visceral hypersensitivity through PAR2-mediated mechanisms (Cattaruzza et al., 2011). However, whether activation of neuronally-expressed PAR2 by CatS results in activation of neurons, sensitisation of TRP channels and transmission of itch signals has not been fully addressed.

The aim of this study was to investigate whether CatS could act directly on neurons to activate them and result in transmission of itch signals. We first established a mouse model of CatS-induced itch to investigate whether TRPV1 and TRPA1 were required for scratching following intradermal injection of CatS. The responses of cultured dorsal root ganglion (DRG) cells to application of CatS were then investigated and the neurons that responded to CatS were characterised.

## 2 Materials and methods

### 2.1 Animals

Experiments were performed in accordance with the United Kingdom Home Office Regulations (Scientific Procedures Act, 1986) in adult male C57BL/6 mice, 12–17 weeks of age. Mice were housed in the Biological Services Unit, King’s College London, and maintained in a 12 h day/night cycle, with access to food and water *ad libitum*. Wild-type mice were obtained from Charles River while TRPV1^−/−^, TRPA1^−/−^ and TRPV1^−/−^/TRPA1^−/−^ mice were a gift from Professor Stuart Bevan, King’s College London. Animals were allowed acclimatisation for 7 days before experiments and habituated for 30 min a day at least 3 days prior to the experiment and on the day of the study.

### 2.2 Behaviour

At least 24 h prior to experiments an area at either the nape of the neck (1.5 cm^2^) or the cheek was shaved in preparation to intra-dermal injection of pruritic agents (50 or 10 μl total volume using a 0.3 ml Myjector 29G insulin syringe) and mice were habituated to the observation chamber for 30 min. Experimenters were blind to genotype and treatment.

In the nape of the neck model, mice were monitored for scratching behaviour immediately post pruritic agent injection for 15 min. A scratch was defined as a rapid back-and-forth lifting of the hind paw to the injected area. Both the time spent scratching (seconds) and number of paw lifts (itching bouts) were recorded and data expressed as time spent scratching and number of foot lifts. The composite score (the two readings added together) was quantified over the 15 min and at 5 min intervals.

In the cheek injection model (Shimada and LaMotte, 2008), scratching was defined as a continuous movement of the hind paw at the site of injection, while wiping was defined as a stroking movement of the forepaw over the injected portion of the cheek. Total scratching and wiping behaviour and scratching over the 15 min observation period were quantified.

### 2.3 Materials and compounds

Human recombinant CatS (hr-CatS), LHVS (morpholinurea-leucine-homophenylalanine-vinylsulfone-phenyl (Barclay et al., 2007) and MDV-590 *((S)-N-(*1-((1-(2-amino-2-oxoacetyl)cyclobutyl)amino)-3-(1-fluorocyclopentyl)-1oxopropan-2-yl)3,3,3-trifluoro-2,2-dimethylpropanamid) (Sohlenius-Sternbeck et al., 2017) and a close analogue of the selective inhibitor MIV-247 (described in Hewitt et al., 2016) were supplied by Medivir, Huddinge, Sweden.

Buffer exchanged, de-salted and heat inactivated CatS or activated hr-CatS (activity 46 µmol AMC (7-amino-4-methylcoumarin) produced/minute/mg; concentration of 0.38 mg/ml by active site titration) (Hewitt et al., 2016) was injected at doses of 1–20 µg/50 µl/mouse-which was the highest deliverable dose due to solubility in saline (vehicle). LHVS was dissolved in 20% cremophore/saline and injected subcutaneously at a dose of 30 mg/kg, 1 h before intradermal hr-CatS. MDV-590 was supplied pre-formulated in 20% HP-β-CD (2-hydroxypropyl)-β-cyclodextrin), at 40 mM concentration and administered via oral gavage at a dose of 200 µmol/kg, 1 h before intradermal hr-CatS. The PAR2 agonist SLIGRL-NH_2_ (10, 50 and 100 µg/50 µl/mouse, (Insight Biotechnology, Wembley, United Kingdom) and antagonist FSLLRY-NH_2_ (50 µg/50 µl/mouse) (Sigma) as well as chloroquine sulphate (200 μg/10 μl/mouse) and capsaicin (10 μg/10 μl/mouse) (Sigma) were dissolved in saline and injected intradermally.

### 2.4 Calcium imaging in cultured dorsal root ganglia

Mice were killed by an overdose of sodium pentobarbital, and dissociated DRG were prepared as reported previously (Simeoli et al., 2017). DRGs from all spinal levels were collected and placed in a dish of Ham’s DMEM F-12 and treated with type-4 collagenase (Worthington, Lorne Laboratories, Reading, United Kingdom; 0.125% w/v) for up to 120 mins at 37 °C. Following trituration, cells were plated on glass coverslips pre-coated with poly-L-ornithine and collagen and incubated for 18–24 h at 37 °C.

Cultured DRG neurons were loaded with Fura-2-AM (2 μM; Teflabs, Cambridge Bioscience, Cambridge, UK) for 60 min at 37 °C in Hank’s balanced salt solution (with calcium and magnesium and without phenol red, Thermo Fisher, Loughborough, UK) supplemented with 10 mM HEPES (Sigma). Coverslips were mounted in an open chamber and substances were applied by continuous perfusion and hr-CatS was incubated for up to 20 min. In some experiments, cells were pre-incubated in MDV-590 (0.5 μM) or FSLLRY-NH_2_ (5 μM) for 10 min followed by application of hr-CatS in the presence of MDV-590 or FSLLRY-NH_2_. The fluorescence of individual cells was measured at 340 and 380 nm excitation and 510 nm emission with a Flexstation (Molecular Devices, Wokingham, UK) using PTI Easy Ratio Pro software (version 1.2.1.87) every 2 s. KCl (Sigma; 50 mM) was applied to all cells at the end of each experiment to provide a maximal Ca^2+^ signal against which to normalise responses to other substances and activate all viable cells to allow for comparisons of the percentage of cells that responded to activation of each substance. A cell was considered to have responded to application of a substance if the response to that substance was at least 20% of the size of the response of the same cell to application of KCl.

### 2.5 Data and statistical analyses

The time spent scratching (seconds) and the number of itching bouts were expressed separately, and also combined in order to provide a composite itch score during a 15 min observation period. Data are expressed at mean + SEM. Time course data were analysed using Two-way ANOVA followed by Dunnett’s test. Combined data over the 15 min observation period were analysed using One-Way ANOVA followed by Dunnett’s test. Post-treatment combined data were analysed with paired/unpaired Student’s *t* test and Mann-Whitney *U* test. Calcium imaging data were analysed using GraphPad Prism 5 and are presented as the mean ± SEM. n represents the number of experiments analysed. Statistical analyses were conducted using one-way ANOVA followed by Dunnett’s multiple comparison test *versus* 20 min buffer incubation to determine the percentage of cells that responded to application of substances.

## 3 Results

### 3.1 Acute injection of cathepsin S induces scratching behaviour

**Fig. 1:**
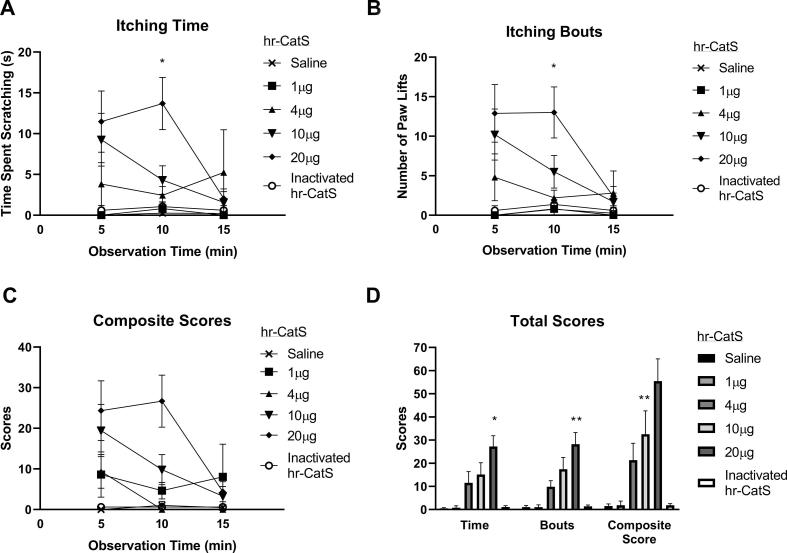
Activated recombinant hr-CatS induces itch-like behaviour. A) Itching time (time spent scratching) over the 15 min observation period after intradermal injection in the nape of the neck. B) Number of paw lifts (Itching bouts) over the 15 min observation period. C) Composite scores over the 15 min observation period. D) Total sum behaviour over the 15 min observation period. Data are mean + SEM of 10 mice per group. *P < 0.05, **P < 0.01, Two-way ANOVA followed by Dunnett’s test *versus* 1 µg in panels A, B and C. One Way ANOVA followed by Dunnett’s test *versus* 1 µg dose in composite behaviour (panel D).

**Fig. 2:**
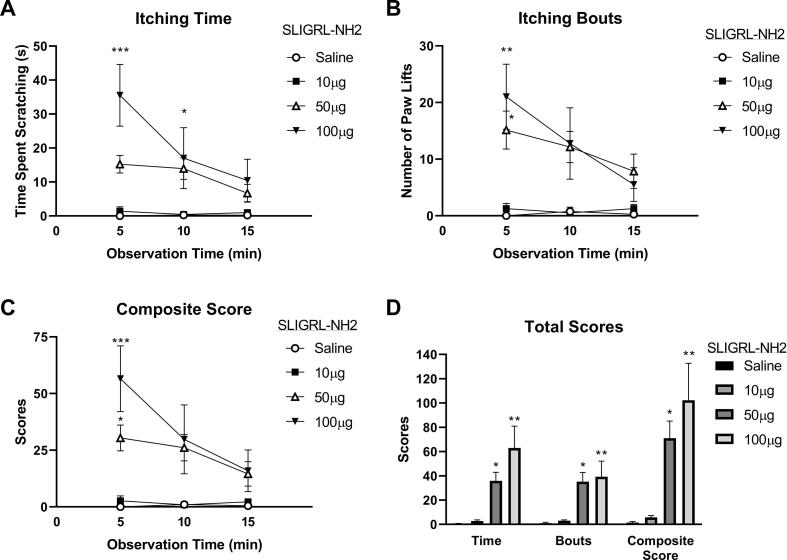
SLIGRL-NH2 induces itch-like behaviour. A) Itching time (time spent scratching) over the 15 min observation period after intradermal injection in the nape of the neck. B) Number of paw lifts (Itching bouts) over the 15 min observation period. C) Composite scores over the 15 min observation period. D) Total sum behaviour over the 15 min observation period. Data are mean + SEM of 7 mice per group *P < 0.05, **P < 0.01,***P < 0.001, Two-way ANOVA followed by Dunnett’s test *versus* 10 µg in panels A,B and C. One-Way ANOVA followed by Dunnett’s test *versus* 10 µg dose in panel D.

Intradermal injection of hr-CatS (1–20 μg/mouse) at the nape of the neck induced significant scratching behaviour compared to saline injection, as indicated by the time spent scratching the neck area during a 15 min observation period (Fig. 1A), number of paw lifts towards the neck (itching bouts, measured as scratching-which it is assumed are in response to an itch) (Fig. 1B), composite scores obtained by combining time and itching bouts (Fig. 1C) and total behaviour scores (Fig. 1D). All itching time and itching bouts behaviour parameters reached statistical significance at 10 min after injection of 20 μg of hr-CatS and ceased by 15 min after injection (Fig. 1A–D). The injection of heat inactivated hr-CatS (20 μg/mouse) did not induce scratching behaviour (Fig. 1 A-D). As expected, the PAR2 receptor agonist SLIGRL-NH_2_ (10–100 μg/mouse) also induced scratching behaviour following intradermal injection at the neck (Fig. 2A–D). At 5 min after 50 or 100 μg/mouse, SLIGRL-NH_2_ injection was associated with significant time spent scratching (Fig. 2A) and significant paw lifts towards the neck (itching bouts) (Fig. 2B), which resulted in significant composite scores (Fig. 2C) and total behaviour scores (Fig. 2D). Total SLIGRL-NH_2_ itch-like behaviour values were nearly double those associated with CatS (102.2 ± 30.5 vs. 55.4 ± 9.6 respectively), which nevertheless produced significant pruritic effect at the highest deliverable dose according to solubility in saline (vehicle).

**Fig. 3:**
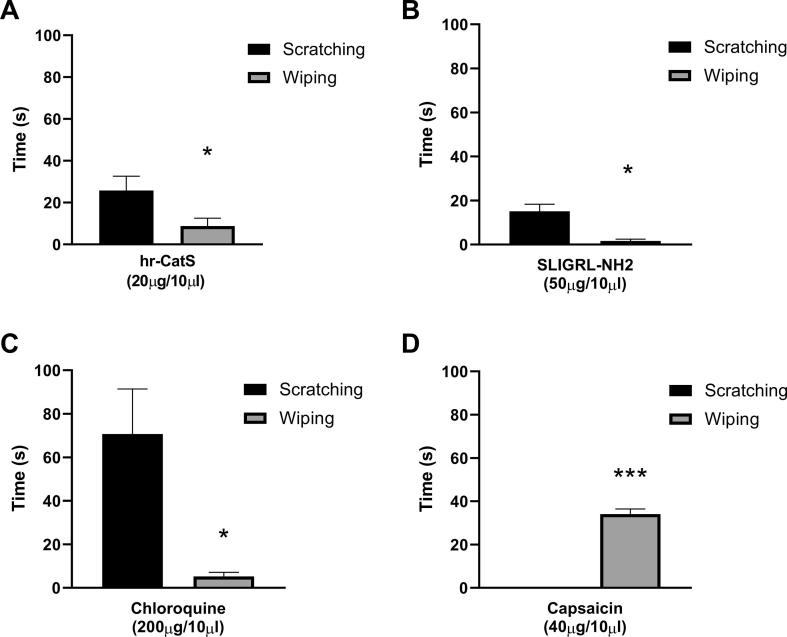
CatS and SLIGRL-NH2 induce itch-like behaviour in the cheek injection model. A) Total time spent scratching or wiping on the cheek over the 15 min observation period immediately after injection of activated hr-CatS. B) Total time spent scratching or wiping on the cheek immediately after injection of SLIGRL-NH_2_. C) Total time spent scratching or wiping on the cheek immediately after injection of chloroquine. D) Total time spent scratching or wiping on the cheek immediately after injection of capsaicin. Data are mean + SEM of 6 mice per group. * P < 0.05,***P < 0.001, Student’s *t* test.

As local injection of hr-CatS in peripheral sites (intraplantar) is known to exert a pro-nociceptive effect (Barclay et al., 2007, Zhao et al., 2014), we differentiated CatS-induced itch-like behaviour from pain-like behaviour using the cheek injection model in which wiping (pain-related) and scratching (itch-related) behaviour can be recorded. We observed that hr-CatS (20 μg/mouse) induced wiping behaviour, which lasted for less than 10 s, but also scratching behaviour, which lasted for an average of 25 s over the 15 min-observation period (Fig. 3A). As expected, the injection of saline resulted in hardly any wiping or scratching behaviour (as shown in Figs. 1 and 2). The injection of SLIGRL-NH_2_ into the cheek also resulted in scratching, but no wiping behaviour (Fig. 3B) and this pattern was observed with the itch agent, chloroquine (Fig. 3C) whilst the injection of the pro-nociceptive agent capsaicin into the cheek was associated with wiping behaviour and no scratching (Fig. 3D). These experiments in the cheek injection model confirm that intradermal CatS, which induces itch sensation in humans, is also a pruritic agent in the mouse. Thus, we examined whether CatS-induced itch was blocked by CatS inhibitors and/or by a PAR2 receptor antagonist as there is evidence for a role of the PAR2 receptor in human studies.

### 3.2 Cathepsin S-induced scratching behaviour is prevented by cathepsin S inhibitors and a PAR2 receptor antagonist.

**Fig. 4:**
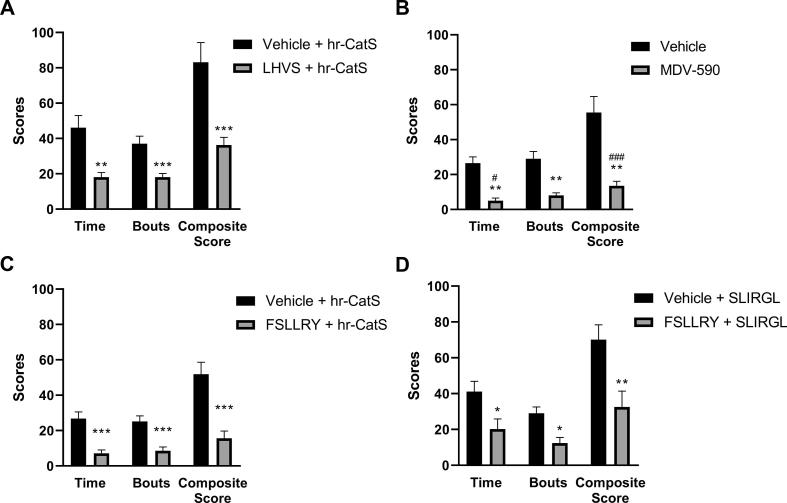
CatS inhibitors and PAR2 antagonist prevent activated Cat-S induced itch-like behaviour. A) Total sum behaviour over the 15 min observation period after intradermal injection of activated hr-CatS (20 µg/mouse) and 1 hr pre-treatment with LHVS (30 mg/kg s.c.). B) Total sum behaviour over the 15 min observation period after intradermal injection of activated hr-CatS (20 µg/mouse) and 1 hr pre-treatment with MDV-590 (200 µmol/kg p.o.). C) Total sum behaviour over the 15 min observation period after intradermal injection of activated hr-CatS (20 µg/mouse) and 1 hr pre-treatment FSLLRY (50 µg/ mouse, intradermal). D) Total sum behaviour over the 15 min observation period after intradermal injection of SLIRGL (50 µg/mouse) and 1 hr pre-treatment FSLLRY (50 µg/ mouse, intradermal); *P < 0.05, **P < 0.01 ***P < 0.001, One Way ANOVA followed by Dunnett’s test *versus* vehicle group. Data are mean + SEM of 8 animals per group.

Systemic administration of the irreversible CatS inhibitor LHVS (30 mg/kg s.c.), 1 hr before intradermal hr-CatS, significantly reduced itch-like behaviour, which was recorded as time spent scratching, number of itching bouts and the composite score (Fig. 4A). Similarly, administration of the reversible CatS inhibitor MDV-590 (200 μmol/kg p.o.) 1 h before hr-CatS significantly reduced time spent scratching, number of itching bouts and the composite score (Fig. 4B). These data indicate that enzymatic activity is required for the behavioural effect associated with administration of CatS. Then, as we postulated that PAR2 receptors expressed by primary afferent fibres could be substrate for CatS, we tested the effect of systemic pre-treatment with a PAR2 receptor antagonist. We observed that FSLLRY (50 μg/kg i.d.) prevented CatS-induced scratching behaviour (Fig. 4C) and also SLIGRL-NH2 -induced itch-like behaviour (Fig. 4D), suggesting that PAR2 receptor activation mediates the effect of CatS.

### 3.3 CatS-induced scratching behaviour requires TRPV1 receptor

**Fig. 5:**
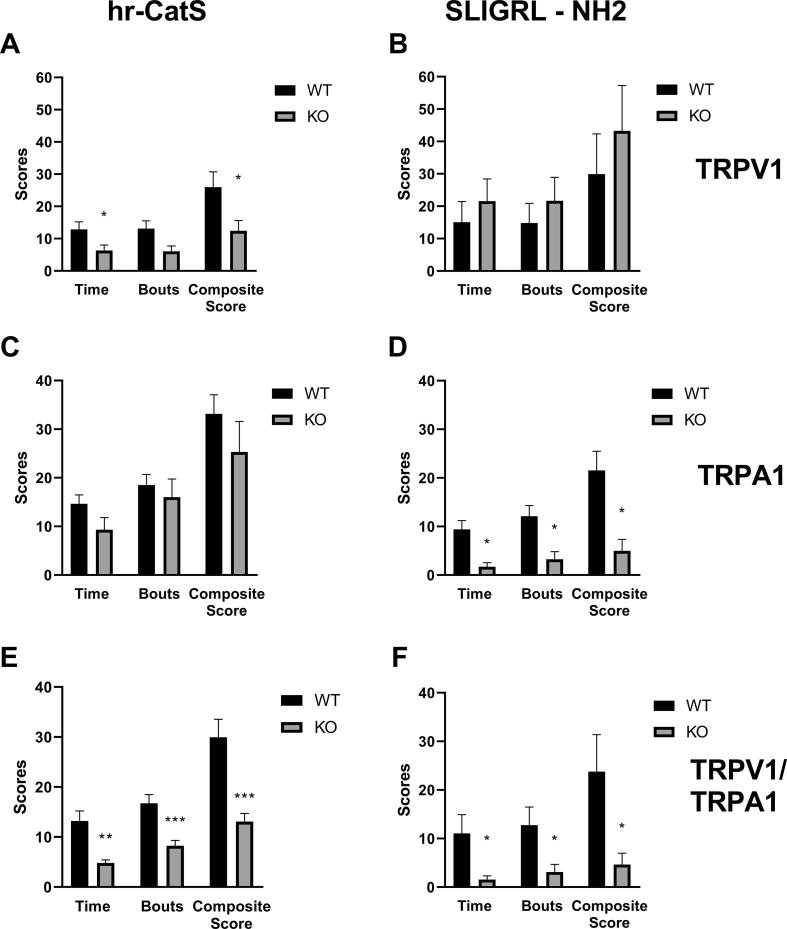
Cat-S and SLIGRL-NH2 induced itch-like behaviour in TRPV1^−/−^ and TRPA1^−/−^ mice. A) Total sum behaviour over the 15 min observation period after intradermal injection of activated hr-CatS (20 µg/mouse) or SLIRGL-NH_2_ (50 µg/mouse). *P < 0.05, **P < 0.01 ***P < 0.001, One Way ANOVA followed by Dunnett’s test *versus* vehicle group. Data are mean + SEM of 8 animals per group.

As cleavage of PAR2 by proteases results in the sensitisation of TRP channels in sensory neurons, we assessed CatS-induced itch-like behaviour in TRPV1^−/−^, TRPA1^−/−^, TRPV1^−/−^/TRPA1^−/−^ double transgenic mice. For comparison purposes we also evaluated SLIGR-NH2-induced itch-like behaviour in the transgenic mice. We observed that in TRPV1^−/−^ mice, hr-CatS-induced scratching behaviour was significantly reduced as time spent scratching, number of itching bouts and the composite score were lower than in WT mice (Fig. 5A). However, SLIGR-NH2-induced itch-like behaviour was unaltered in TRPV1^−/−^ mice, which showed comparable time spent scratching, number of itching bouts and the composite score to WT controls (Fig. 5B). In contrast, hr-CatS-induced scratching behaviour was unaltered in TRPA1^−/−^ (Fig. 5C) whereas SLIGRL-NH2-induced scratching behaviour was reduced by 80% (Fig. 5D). Predictably, in TRPV1^−/−^/TRPA1^−/−^ mice, both CatS- and SLIGR-NH2-induced itch-like behaviour was significantly reduced compared to WT mice (Fig. 5 E, F). This set of data suggests that TRPV1 is required for CatS-induced itch and confirm the requirement of TRPA1 receptor for SLIGRL-NH2-mediated itch.

### 3.4 Sensory neurons respond to CatS

**Fig. 6:**
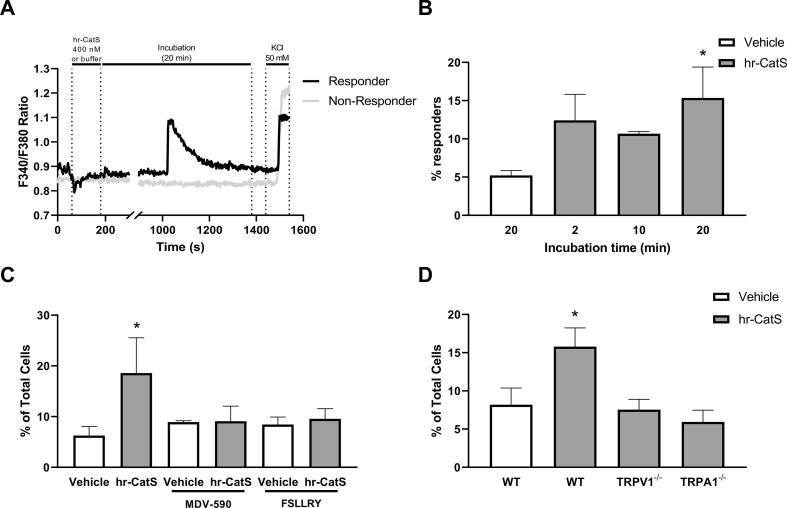
Calcium responses in sensory neurons following application of CatS are reduced by the PAR2 antagonist and in cultures from TRPV1^−/−^ or TRPA1^−/−^ mice. A) Representative traces of the calcium response of a cell that responded to application of hr-CatS (400 nM). No changes were observed between 200 and 1000 s and this is reflected in the break of the axis. B) Percentage of sensory neurons in culture that responded to buffer or hr-CatS applied for up to 20 min, from 461 cells, n = 4, 5 from 1 coverslip per mouse. C) Percentage of sensory neurons in culture that responded to application of hr-CatS in the presence of MDV-590 (0.5 µM) or FSLLRY (5 µM), from 663 cells, n = 3, 4 from 1 coverslip per mouse. D) Percentage of sensory neurons cultured from TRPV1^−/−^ or TRPA1^−/−^ mice that responded to application of CatS, from 373 cells, n = 3–5 from 1 coverslip per mouse. *P ≤ 0.05, One Way ANOVA followed by Dunnett's test *versus* vehicle incubation. Data are mean + SEM.

To examine whether sensory neurons could respond directly to the application of CatS, cultured mouse DRG neurons were incubated with hr-CatS, which induced an increase in intracellular calcium concentration following incubation at 400 nM for 20 min in cells that subsequently responded to KCl incubation (Fig. 6A). Specifically, more than 15% of cells responded to hr-CatS, which was significantly greater than the percentage that responded to application of buffer for the same duration (Fig. 6B). Some cells also responded following exposure to hr-CatS for 2 or 10 min, although this was not significantly different compared with the percentage that responded to buffer (Fig. 6B).

When DRG neurons were incubated with the CatS inhibitor MDV-590 for 10 min prior to hr-CatS, the percentage of cells that responded to hr-CatS was reduced by more than 50% compared to the percentage that responded to hr-CatS in the absence of the inhibitor, and was similar to the percentage that responded to buffer (Fig. 6C). Similarly, incubation of DRG neurons with the PAR2 antagonist FSLLRY-NH2 (10 min prior to hr-CatS) also resulted in a significant reduction of the percentage of cells that responded to hr-CatS (Fig. 6C). These data suggest that PAR2 receptors mediate CatS-induced calcium fluxes in DRG neurons.

Finally, in order to test for the involvement of TRPV1 and TRPA1 channels on the effect of CatS we used DRG neurons from TRPV1^−/−^ and TRPA1^−/−^ mice and observed that the percentage of cells responding to hr-CatS was reduced in both conditions (Fig. 6D).

## 4 Discussion

In agreement with previous observations in humans (Reddy et al., 2010), we report that CatS is a pruritogen eliciting scratching behaviour when injected into mice. CatS-induced scratching was prevented by CatS inhibitors and PAR2 antagonist suggesting that PAR2 enzymatic activation mediates CatS-induced itch. In addition, CatS-induced scratching was reduced by 50% in TRPV1^−/−^ mice, supporting a role for TRPV1 in CatS-induced itch. In contrast, scratching behaviour was not significantly reduced in TRPA1^−/−^ mice, suggesting this channel may not be required for CatS-induced scratching. The opposite was observed for SLIGRL-induced scratching behaviour, with TRPV1 appearing to be dispensable for SLIGRL-induced behaviours while expression of functional TRPA1 was required.

Application of CatS to DRG neurons in culture resulted in intracellular calcium increase, suggesting neurons can respond directly to CatS. The percentage of cells that responded to CatS is similar to the ∼7% of mouse DRG neurons that were reported to respond in a previous study (Reddy et al., 2015), with the majority of responding cells in our study showing an increase in intracellular calcium within 2 min of CatS application.

Responses to CatS were prevented by the CatS inhibitor, MDV-590, confirming the calcium fluxes observed were due to the enzymatic activity of CatS. The effect of CatS was also prevented by the PAR2 antagonist FSLLRY-NH_2_, suggesting that PAR2 mediated the effects of CatS. Moreover, the percentage of cells that responded to CatS was comparable to the percentage of mouse DRG cells in culture reported to express PAR2 mRNA (Vellani et al., 2010). However, the observation that CatS and SLIGRL-induced scratching behaviours had different time courses and were differentially affected in transgenic mice lacking functional TRPV1 or TRPA1 suggests CatS and SLIGRL may work via different mechanisms. For instance, SLIGRL can activate PAR2 immediately on contact, whereas the CatS would need time for the proteolytic cleavage. It has recently been reported that CatS, SLIGRL-NH_2_ and mucunain can act via MrgprC11 in addition to PAR2 (Liu et al., 2011, Reddy et al., 2015, Reddy et al., 2018). One possibility is that activation of sensory neurons *in vivo* by CatS or SLIGRL-NH2 results in differential activation of these receptors, which could explain our observations. In contrast, preventing activation of PAR2 *in vitro* appears to be sufficient to prevent CatS-induced calcium responses in DRG neurons. The finding that CatS-mediated calcium transients can be reduced in the presence of a PAR2 antagonist is not in agreement with previous reports that CatS-mediated calcium fluxes were not abolished in DRG cultures from PAR2^−/−^ mice (Reddy et al., 2015). However, CatS at 2–5 μM concentration was used in these experiments, while we used CatS at a lower concentration (400 nM). It cannot be excluded that CatS when used at higher concentrations could activate additional receptors, such as Mrgprs, while PAR2 could be preferentially cleaved with lower concentrations of CatS. Several *in vitro* and *in vivo* observations indicate that human CatS can disarm mouse PAR2 receptor: i) human and mouse PAR2 receptors share more than 80% identity (Bohm et al., 1996); ii) hr-CaS cleaves mouse PAR2 receptors at two sites (Elmariah et al., 2014, Zhao et al., 2014) and ii) PAR2 antagonists block intraplanar hr-CatS-induced pain in mice (Zhao et al., 2014).

CatS-induced calcium responses were also reduced in DRGs cultured from TRPV1^−/−^ or TRPA1^−/−^ mice, supporting a role for these channels in activation of sensory neurons by CatS and the subsequent transmission of itch signals. This is in agreement with the notion that pruriceptors belong to a subset of TRPV1 and TRPA1-expressing neurons (Liu and Ji, 2013). Activation of these receptors results in the opening of channels to form a pore allowing the passage of cations including calcium and sodium. The calcium fluxes observed following activation and opening of these channels can be prevented by the removal of extracellular calcium from the buffer, suggesting that most or all of the calcium is derived from the extracellular environment (Caterina et al., 1997, Jordt et al., 2004).

The observation that calcium responses to CatS *in vitro* required expression of both TRPV1 and TRPA1 channels is surprising given that TRPA1 was shown to be dispensable for CatS-induced scratching behaviour. One explanation for this is the difference between *in vivo* behavioural studies and *in vitro* assays used to investigate the responses of cells, which may not necessarily be the same; in this instance we are assuming that calcium fluxes and activation of cells means transmission of itch signals and subsequent scratching behaviour. Compensatory mechanisms may exist in TRPA1^−/−^ mice such that no obvious defects in CatS-mediated scratching are observed. For instance, it has been reported that CFA-induced hyperalgesia is reduced in wild-type mice pre-treated with a TRPA1 inhibitor, while TRPA1^−/−^ mice still displayed hyperalgesia (Petrus et al., 2007). Changes in the expression of other TRP channels in DRGs and other cell types are proposed to be responsible for this, and a similar compensatory mechanism may allow for CatS-induced scratching behaviour in TRPA1^−/−^ mice, even in the absence of calcium responses in DRGs *in vitro*. Alternatively, because TRPV1 and TRPA1 are also reported to be expressed on skin keratinocytes and mast cells as well as sensory neurons (Atoyan et al., 2009, Bíró et al., 1998, Inoue et al., 2002), the presence or absence of these channels on non-neuronal cells could also be mediating an indirect effect on CatS-induced scratching in behavioural studies which would not be detected in neuronal calcium imaging experiments. Thus, although CatS did not cause calcium responses in neurons cultured from TRPA1^−/−^ mice, itch sensations and scratching behaviour might still occur, for instance, via release of histamine from mast cells and sensitisation or compensation of TRPV1 expressed on neurons or other cells. Since PAR2 sensitises TRPV1 and TRPA1, we hypothesise cleavage of neuronal PAR2 by CatS is responsible for this sensitisation. Cleavage of PAR2 by trypsin has previously been reported to sensitise TRPV1 via phosphorylation by protein kinases C and A, and PAR2-mediated sensitisation occurs by hydrolysis of PIP_2_ by PLC (Amadesi, 2004, Amadesi et al., 2006, Dai, 2004, Dai et al., 2007). However, whether CatS causes sensitisation of neuronally-expressed TRPV1 and TRPA1 by the same mechanisms is not yet known. It would therefore not be so surprising if the mechanisms by which TRPV1 and TRPA1 became sensitised by PAR2 were the same following cleavage of PAR2 by CatS compared with trypsin.

In summary, CatS is a pruritogen that causes scratching behaviour when injected in mice and activates sensory neurons in culture. TRPV1 is required for both CatS-induced scratching behaviour and CatS-mediated calcium fluxes *in vivo*. As CatS inhibitors are effective at preventing CatS-induced itch, this model can be used as a translational model as well as for testing new indications for CatS inhibitors.

## Competing interest statement

The authors have no competing interests to declare. Ellen Hewitt and Erik Lindstrom are former Medivir employees.
